# Medical and Physical Intelligence

**Published:** 1807-03

**Authors:** 


### MEDICAL AND PHYSICAL INTELLIGENCE.

*Domestic Origin of Hooping-Cough.* By Dr. A. C. Willey, of Block-Island, America.

The tussis convulsiva, or hooping-cough, occurred here in April, 1805, and did not become wholly extinct till some time in autumn. What rendered it particularly worthy of attention, was its being indigenous. It made its appearance over the greater part of the island at the same time, and was untraceable to any apparent source. The insulated situation of this place is extremely favourable to observations, and the detection of facts of this nature, without the danger of deception; and has afforded, in the present instance, a fair demonstration that the hooping-cough can originate without contagion. Indeed I am inclined to believe, that the rise and progress of this epidemic disease does not depend so much upon contagion as is generally imagined. The universal belief that the system, during the operation of *pertussis,* generates a specific virus, capable of communicating the disease, seems to have prevented the mind from looking any further for a principle adequate to its production. But because the living system possesses the power of elaborating this virus, I know of no reason why we should deny its formation in the departments of inanimate nature. The laws of physiology and inorganic matter agree in the production of soda and lime; why not then in the generation of pertussic poison? That there *is* a principle, independent of contagion, capable of inducing this complaint, I feel fully convinced, not only from its origination on this island, but from the number of cases which I have seen in those who have never been exposed to contagion. This principle undoubtedly exists in the atmosphere, which it pervades to a certain extent; but what it is, and how formed, remains a curious subject for physical research.

### *Use of volatile Alkali as a Counter-Poison.*

Mr. Dubernard, of France, has given an account of the successful treatment of three persons bitten by mad dogs. He describes his method in the following terms: "The wounds were dilated and well washed with a strong solution of sea-salt, and afterwards seared with a red-hot iron. Immediately after, a blistering plaster was applied to each of the wounds. The suppuration was copious, and kept up for a month by gentle epispastics. After the first dressing, the patient was put upon the use of volatile alkali. This he took three times a day, for a fortnight, to the amount of fifteen drops in two ounces of water. After this the dose was altered to twenty drops twice a day, morning and night, night, and continued until the thirty-fourth day from the accident. It was then discontinued, having never been intermitted but twice during the whole time, and that in the morning, for the purpose of taking mercurial pills. On the forty-fourth day from the bite, the patient went about his usual business."

Another person bitten by the same dog, and not treated according to Mr. Dubernard's plan, died on the 26th day, with all the symptoms of confirmed rabies.

The Medical Society of the department of Gers, where Mr. D. practises, has expressed an opinion on the treatment in the following words: "The treatment employed by Mr. Dubernard is predicated upon correct principles. According to the theory and experience of the best practisers, it is a matter of primary consequence to destroy the hydrophobic virus by the actual cautery. With the same intention he has subjected to a long suppuration, the flesh tainted with the venom, that it may be freed from every vestige of it. But he has not stopped there. His prudence taught him to prescribe internally the remedies proper to subdue the poison which might have slipped into the channels of the circulation; and by these means he had enjoyed the satisfaction of seeing the individuals whom he has under his care entirely preserved from the canine madness."

---

"*Efficacy of Poke-Root.* By NATHAN CRAWFORD, of Columbia County, in America.

"IN the year 1797, a young woman, twelve years old, was bitten by a dog in canine madness. About twelve months afterwards she complained of much distress, and said she was 'going mad.' Her hands and feet were cold and clammy, and her countenance quite pale. Her fits came on regularly twice in twenty-four hours, and lasted about one hour each time. If cats or dogs, to which she had an utter abhorrence, came in her sight, they never failed to bring on the fits. In the fits she had a prodigious strength; so that three people were required to keep her in the house. It did not appear she had that aversion to fluids that is common. She had been so ill, that her friends had made her a coffin, when a travelling man prescribed as much poke-root (Phytolacca decandra), rubbed into a powder, as would lay on the point of a case-knife, infused into a gill of new milk, which dose was to be repeated three times a day. She found an alleviation of the symptoms after a day or two, and, by continuing the medicine, she was restored to perfect health, and has continued free from any symptoms of it ever since. It appears to me the above case confirms Dr. Mease's theory of this terrible distemper more than any thing I have heard of. It shows the strong analogy between hydrophobia and tetanus. The poke-root had an operation on her stomach and bowels, analogous to the operation operation of the tincture of cantharides given to the tetanic patient by Dr. Brown of Kentucky. I am sorry that I can bring no proofs of the efficacy of the poke-root in addition to the above, but have sent it forward, hoping it may prove beneficial to our fellow mortals in similar situations."

---

The following account of the very singular consequences of the bite of a rattle-snake, is equally curious and interesting. In the summer of 1801, Mrs. Alfred Beeman, of Luzerne county, in Pennsylvania, was bitten by a rattle-snake. She was then in the fourth or fifth month of her pregnancy. Notwithstanding the alarming symptoms commonly attending the bite of that animal, Mrs. Beeman recovered, and was delivered without accident at the usual time. The child seemed healthy; but no sooner did it begin to suck, than it turned quite black like the snake, swelled considerably, and soon died. A puppy was then procured to draw the breast; the animal died in two days, with the same symptoms. A lamb was next tried; and then a dog, and three other lambs successively, which all shared the fate of the child. A third dog was then procured; it was attacked with slight symptoms of disease, but survived. The mother continued in good health. Two years afterwards, Mrs. Beeman brought into the world another child; apprehensive of losing it like the former, she sent for Dr. Barstow, who in consequence of the long interval which had taken place since the bite, and the recovery of the last dog which had sucked her, prevailed upon her to suckle her child, which was attended with no ill consequence whatever.

At a late meeting of the first Class of the National Institute, M. Hauy, among other papers, read a Report on the Galvanic phenomena discovered by M. Ermann, a member of the Academy of Berlin, for which the annual prize founded by the Emperor was adjudged to that philosopher. The Galvanic Society has, by repeated experiments, ascertained two curious phenomena; namely, 1. That distilled water, subjected to the galvanic action, evidently undergoes a change in its state in a vessel in which oxygen is disengaged by a conducting wire, communicating with the positive pole. That water, in this new state, invariably exhibits the real characteristics of muriatic acid.

The reputation of Dr. Gall, the craniologist, seems to be on the decline in Germany. At Munster, Cologne, Franckfort, and other places, he was not able to collect a sufficient number of subscribers for a course of lectures; and his system is now deemed in his own, as well as other countries, one of the most absurd and visionary that ever presented itself to the credulity of mankind.

There are few countries in Europe where vaccination has made such a rapid and general progress as in the Danish dominions. The committee, which was appointed to facilitate its propagation, receive every day intelligence of its being extended to the most distant tant tant parts of the monarchy, the islands of Ferroe, Iceland, and even Greenland. In 1802, the number of persons vaccinated was only 6,849; but in 1805, it amounted to 23,185.

Dr. CLARKE and Mr. CLARKE will begin a Course of their Lectures on Midwifery, and the Diseases of Women and Children, on Thursday March the 26th. The Lectures are read every day at the house of Mr. Clarke, No. 10, Upper John Street, Golden Square, at a quarter past ten o'clock in the morning, for the convenience of Students attending the Hospitals. For Particulars apply to Dr. Clarke, New Burlington Street, or to Mr. Clarke, No. 10, Upper John Street, Golden Square.

Dr. H. ROBERTSON, of Edinburgh, has in the press a Work on the Natural History of the Atmosphere, and of its Influence in the Success of Medicine and Agriculture, &c. We are likewise informed, that the same gentleman, has nearly ready for publication, a Book, containing Discourses on the Nature of Inflammations and of the History, Theory, and Cause of the Venereal Disease.

A new edition, being the fifth, is in the press, of Dr. BREE'S Inquiry into Disordered Respiration; a work which has continued to establish itself in the public estimation, so as now to rank among our medical classics.

Mr. COOPER, of Golden Square, has in the press a work likely to prove highly useful to the profession at large, and particularly to students, under the title of First Lines of the Practice of Surgery.

Dr. J. E. SMITH proposes shortly to publish an Introduction to Botany, in one volume octavo, with a few plates, intended for the use of female as well as male students of that delightful science, and divested of every thing that might be deemed exceptionable.

---

